# Research advances in the application of betulinic acid for anti-inflammatory and anti-tumour therapy

**DOI:** 10.1186/s13020-026-01424-x

**Published:** 2026-05-26

**Authors:** Zewen Chu, Shuaiyu Jiang, Yuting Li, Xiang Li, Wan Xin Koh, Pottakorn Aruncharoenphonchai, Yue Ding, Yanwei Xiang

**Affiliations:** 1 School of Rehabilitation Science, Shanghai University of Traditional Chinese Medicine, 1200 Cai Lun Road, Zhangjiang Hi-TechPark, Pudong New Area, Shanghai, 201203 China; 2 Institute of Rehabilitation Medicine, Shanghai Academy of Traditional Chinese Medicine, Shanghai, 201203 China; 3 Engineering Research Center of Traditional Chinese Medicine Intelligent Rehabilitation, Ministry of Education, Shanghai, 201203 China; 4 School of Pharmacy, Shanghai University of Traditional Chinese Medicine, 1200 Cai Lun Road, Zhangjiang Hi-TechPark, Pudong New Area, Shanghai, 201203 China

**Keywords:** Betulinic acid, Traditional Chinese medicine, Active compound, Anti-inflammatory, Anti-tumour therapy

## Abstract

**Abstract:**

Betulinic acid (BA) is a naturally occurring pentacyclic triterpenoid widely distributed in a variety of medicinal plants and has attracted considerable attention as an active constituent of several traditional herbal medicines. In recent years, research on BA has increasingly focused on cancer and inflammation owing to its broad-spectrum pharmacological activities against tumours and inflammation-related diseases. This review summarizes recent advances in the study of BA and its derivatives, with particular emphasis on their antitumour and anti-inflammatory properties, underlying molecular mechanisms, and strategies to improve therapeutic efficacy. Accumulating evidence indicates that BA exerts its antitumour and anti-inflammatory effects through multiple pathways and molecular targets. In addition, representative structural modification approaches and nano-enabled drug delivery strategies developed to overcome the intrinsic limitations of BA, such as poor aqueous solubility and low bioavailability, are systematically discussed. Finally, key challenges associated with the clinical translation of BA are highlighted from a translational perspective, with the aim of providing references for further experimental investigations and clinical development. Overall, BA represents a promising bioactive monomer with dual anti-tumour and anti-inflammatory potential, particularly in the context of inflammation-associated cancers.

**Conclusions:**

The research and development prospects of BA and its derivatives as active monomeric compounds in traditional Chinese medicine are promising, yet several challenges remain. In terms of research directions, further structural optimization of these derivatives is needed to enhance their biological activity, selectivity, and pharmacokinetic properties. In addition, exploring new targets and mechanisms of action represents another important avenue, which may provide a more solid theoretical and experimental foundation for the development of BA as a new anti-tumour and anti-inflammatory drug.

**Graphical abstract:**

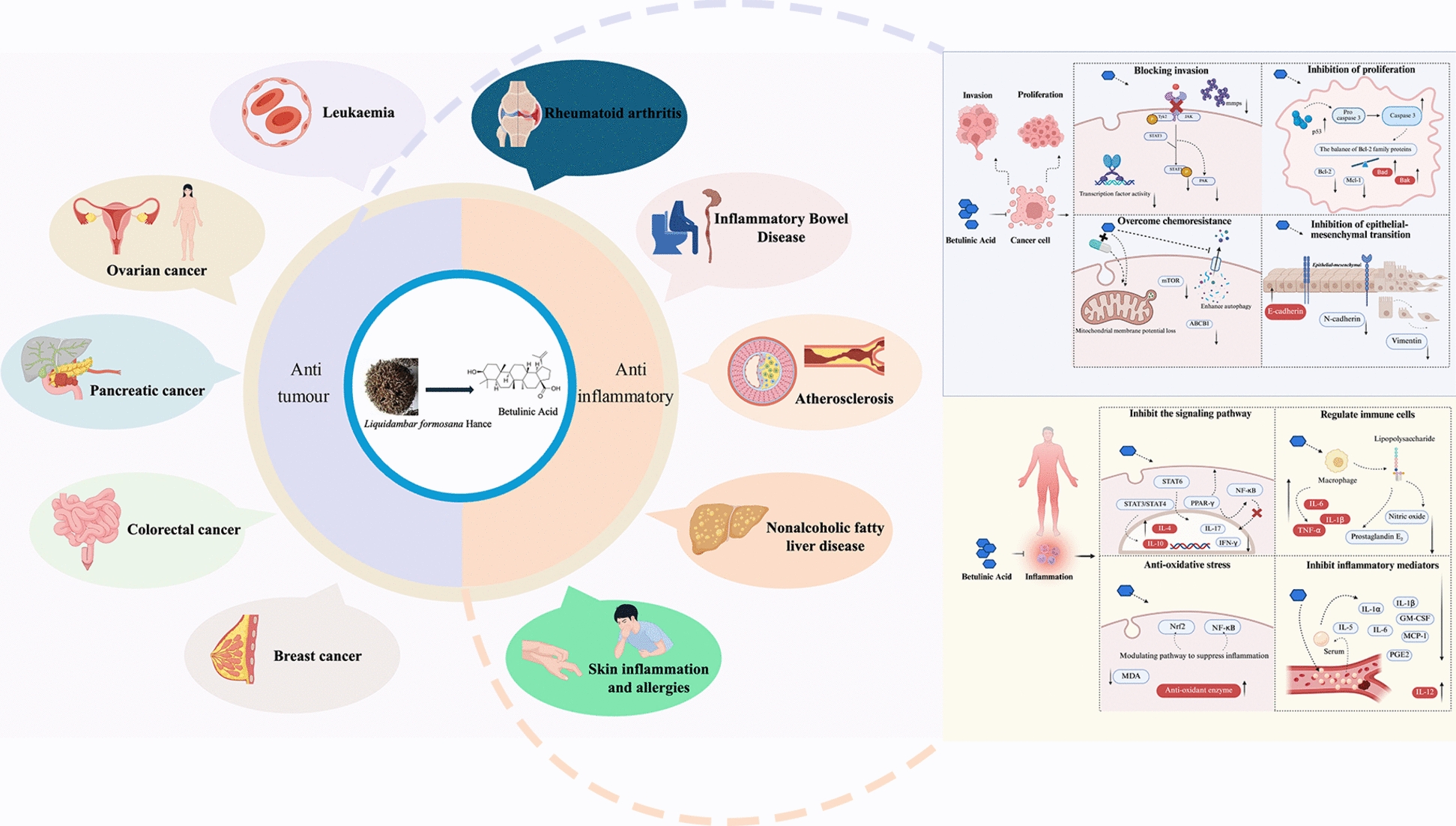

## Background

In recent years, natural compounds have garnered extensive attention in the treatment of cancer and various other diseases. As the largest family of natural compounds, triterpenoids have been studied for their mechanisms and therapeutic effects in cancer treatment [1]. Betulinic acid (BA), a triterpenoid, has attracted particular interest because of its broad-spectrum and potent anti-tumour activity. The primary methods for treating tumours in clinical practice include surgical intervention, radiotherapy, and chemotherapy [2]. However, the high cost of chemotherapeutic drugs and the severe side effects of radiotherapy and chemotherapy significantly impact patients’ treatment experience and quality of life [3]. Consequently, identifying more economical alternative therapies with reduced adverse effects has become a research priority. In this context, natural medicines, particularly traditional Chinese medicine (TCM), have garnered extensive attention because of their potential advantages in tumour treatment [4]. Research has indicated that traditional Chinese compounds such as cinobufotalin, geniposide, icaritin, plumbagin, and Houttuynia cordata have demonstrated favourable outcomes in clinical trials [5]. Furthermore, their active constituents and potential targets have been thoroughly elucidated, providing crucial scientific foundations for anti-tumour drug discovery [6–10]. Research has indicated that natural compounds, through mechanisms involving classical signalling pathways, immune checkpoints and the gut microbiota, have cancer-delaying and therapeutic effects. These findings collectively demonstrate that natural compounds have broad application prospects in cancer therapy [11].

This review presents the latest advances in the application of BA and its derivatives in cancer therapy. For literature screening, we systematically searched databases such as PubMed and Web of Science, covering the period from January 2010 to December 2025. The inclusion criteria were: (1) English-language literature focusing primarily on BA; (2) studies explicitly addressing anti-tumour and anti-inflammatory topics. The exclusion criteria were: (1) non-English publications; (2) conference papers, patents, and unpublished materials. We will examine the anti-tumour pharmacological properties, mechanisms of action, and delivery methods of BA. Furthermore, we investigate the therapeutic efficacy and specific mechanisms of BA and its derivatives against various cancer types, as well as the delivery routes and strategies for BA in cancer treatment.

## Sources, chemical structure, and physicochemical properties of BA

BA, chemically designated as 3β-hydroxy-lup-20(29)-en-28-oic acid (C_30_H_48_O_3_), is a naturally occurring pentacyclic triterpenoid [12]. In 1788, Lowitz first isolated betulin from birch bark [13]. Subsequently, its oxidised derivative, BA, was discovered in 1902 from extracts of Gratiola officinalis [14]. It was not until 1976 that research first reported the anti-tumour activity of BA against the P-388 lymphatic system leukaemia experimental model [15]. BA is an active component in numerous TCM [9], is primarily extracted from the fruits of Liquidambar formosana Hance [16] and is also widely present in various plants, such as lupins, Belamcanda chinensis, and eucalyptus [17]. However, the natural content of BA in source plants is generally low and often insufficient to meet the demands of pharmacological research and pharmaceutical development. Consequently, researchers frequently employ semisynthetic methods to obtain this compound.[16]. Consequently, researchers frequently employ semisynthetic methods to obtain this compound. Among these methods, the selective oxidation of betulin on solid aluminium supports results in high selectivity and rapid reaction rates, achieving yields ranging from 93 to 98% [18].

As a lupane-type phytochemical within the pentacyclic triterpenoid class, BA possesses four six-membered rings and one five-membered ring. It features an isopropyl group at the C-19 position [19]. Its chemical structure comprises five fused rings, exhibiting a distinctive spatial configuration. This structure confers specific physical and chemical properties upon BA. For instance, BA has poor water solubility [20], consistent with the highly lipophilic nature of the lupane-type pentacyclic triterpenoid scaffold. Like its parent compound betulin, BA exhibits limited aqueous solubility, which contributes to its low oral bioavailability[21]. Although BA remains relatively stable under conventional conditions, it may undergo chemical reactions under specific environmental conditions. Environmental mass spectrometry techniques such as desorption atmospheric pressure photoionisation (DAPPI) applied to birch bark have revealed that BA is an important defensive compound within the bark and exhibits a variable distribution across different developmental stages and tissue locations [22]. BA exhibits broad pharmacological activity, despite its efficacy in antitumour, antiviral, anti-inflammatory, antibacterial, and antiparasitic applications [17]. More significantly, research has confirmed that BA can induces apoptosis, exhibiting specific toxicity towards multiple tumour cell lines [21], while exerting minimal effects on normal cells [23]. These properties not only influence the natural occurrence of BA but also significantly impact its application in cancer research, providing a foundation for subsequent structural modification and drug development.

To enhance the antitumour activity of BA and improve its water solubility, numerous structural modification approaches have been explored. These primarily involve modifying the hydroxyl group at C-3 or the C-28 hydroxyl group in betulin or the carboxyl group at C-28 in BA. This approach not only enhances the pharmacokinetic properties of BA but also, in recent studies, through the introduction of diverse functional groups, enables BA and its derivatives to serve as twin drugs alongside the original compound [24]. Research has indicated that BA exerts antidiabetic effects by reducing glucose absorption, decreasing endogenous glucose production, increasing insulin sensitivity, improving lipid homeostasis, and promoting weight homeostasis [25]. With respect to weight homeostasis, BA regulates adipose and carbohydrate metabolism to control body weight. A study demonstrated that BA treatment in high-fat diet-fed mice has potent anti-hyperlipidaemic effects [26]. In summary, the multifaceted pharmacological activities of BA in combating diabetes, lowering blood lipids, and reducing inflammation confer broad therapeutic potential for metabolic syndrome management [27].

## Mechanisms underlying the antitumour effects of BA

### Mechanisms by which BA inhibits tumour cell invasion

Tumour cell invasion constitutes a pivotal stage in the metastatic process. BA inhibits this process through multiple mechanisms. Research has indicated that the BA derivative SYK023 significantly suppresses the metastatic capacity of lung cancer cells [28]. Both in vitro and in vivo experiments demonstrated that low doses of SYK023 effectively inhibited lung cancer cell metastasis by downregulating genes associated with cell migration, such as synaptopodin, thereby suppressing F-actin polymerisation. Furthermore, BA can suppress tumour cell invasion by interfering with the epithelial–mesenchymal transition (EMT) process. In breast cancer studies, BA inhibits migration and invasion by modulating STAT3 and FAK activation, thereby downregulating matrix metalloproteinase (MMP) expression and upregulating the expression of the inhibitor TIMP-2 [29].

At the molecular mechanism level, BA can also inhibit tumour cell invasive behaviour by regulating key signalling pathways. For instance, in human renal carcinoma cells, BA treatment resulted in the upregulation of MMP2, MMP9 and vimentin expression but the downregulation of tissue metalloproteinase inhibitor 2 and E-cadherin expression. This series of changes correlates with a reduction in tumour cell invasive capacity [30]. Moreover, BA can regulate downstream genes associated with invasion by inhibiting the activity of transcription factors such as specific protein 1 (Sp1). Studies in prostate cancer cells have confirmed this mechanism and revealed the pivotal role of Sp1 in this process [31, 32]. In addition, Chu found that betulonic acid, as one of the active monomeric components of the stem of *Celastrus orbiculatus* Thunb, inhibited the invasion and metastasis of gastric cancer cells by regulating cytoskeletal remodeling [33]. It is worth noting that betulonic acid is an oxidized derivative of BA characterized by the conversion of the C-3 hydroxyl group into a carbonyl moiety. This structural modification leads to distinct physicochemical properties and biological activity profiles compared with BA. The aforementioned research fully demonstrates that BA inhibits tumour cell invasive behaviour through multiple pathways, thereby offering a novel potential strategy for preventing and treating tumour metastasis.

### Mechanism through which BA inhibits tumour cell proliferation

BA inhibits tumour cell proliferation through multiple mechanisms, including cell cycle arrest and apoptosis induction. With respect to cell cycle regulation, BA induces cyclostatic effects in tumour cells. For instance, in the human colon cancer cell line HT-29, the dermocarpic acid derivative 2c induces cell cycle arrest at the G0/G1 phase. This mechanism involves downregulating the expression of cyclin D1 and proliferating cell nuclear antigen (PCNA) while simultaneously upregulating p21 expression, ultimately inhibiting cell proliferation [34]. In addition to cell cycle arrest, BA also inhibits tumour cell proliferation through the induction of apoptosis, which is discussed in detail in “Mechanism through which BA induces the apoptosis of tumour cells” section. Furthermore, in human cervical carcinoma HeLa cells, BA induces G0/G1 phase cell cycle arrest by downregulating phosphoinositide 3-kinase (PI3K) subunit expression and inhibiting Akt phosphorylation, thereby suppressing cellular proliferation. The apoptotic effects of BA in HeLa cells via the mitochondrial pathway are further discussed in “Mechanism through which BA induces the apoptosis of tumour cells” section [35]. These findings indicate that BA effectively inhibits tumour cell proliferation primarily through inducing cell cycle arrest at the G0/G1 phase via the regulation of key cell cycle regulators such as cyclin D1, PCNA, p21, and the PI3K/Akt pathway.

### Mechanism through which BA reverses chemotherapy resistance

Chemotherapy resistance represents one of the primary obstacles in clinical oncology treatment, while BA has demonstrated considerable potential in reversing such resistance. Research has indicated that BA is cytotoxic to multiple multidrug-resistant tumour cell lines and acts independently of classic resistance-associated proteins such as P-glycoprotein and breast cancer resistance protein [36]. In the human breast cancer cell lines M.D. Anderson Cancer Center-Metastatic Breast Cancer 231 (MDA-MB-231) and Michigan Cancer Foundation-7 (MCF-7), BA and its derivatives effectively inhibit cell proliferation and migration under both normoxic and hypoxic conditions, induce apoptosis, and simultaneously suppress hypoxia-induced gene expression [37].

At the molecular level, BA can overcome chemotherapy resistance by modulating key signalling pathways. For instance, in studies of lung cancer cells, BA enhances the cytotoxic effects of epidermal growth factor receptor tyrosine kinase inhibitors on Epidermal growth factor receptor Tyrosine kinase inhibitor-resistant cells [38]. Combined treatment enhances the accumulation of cells in the sub-G1 phase, induces the loss of mitochondrial membrane potential and initiates apoptosis while simultaneously affecting cell cycle progression and autophagy-related protein expression. Furthermore, in pancreatic cancer studies, BA enhances tumour cell sensitivity to chemotherapeutic agents by inhibiting mTOR signalling [39]. These findings indicate that BA reverses chemotherapy resistance through multiple mechanisms, offering novel insights for enhancing the efficacy of tumour chemotherapy.

### Mechanism through which BA induces the apoptosis of tumour cells

The core mechanism through which BA induces tumour cell apoptosis is the mitochondrial pathway. In studies on the human colon cancer cell line HT-29, the BA derivative 2c induced apoptosis characterised by elevated intracellular ROS levels, mitochondrial membrane potential depolarisation, sequential activation of caspase-3 and caspase-9, Poly Adenosine diphosphate-ribose polymerase (PARP) cleavage, and increased expression of pro-apoptotic proteins such as Bax and Bad. Concurrently, the expression of the antiapoptotic proteins B-cell lymphoma 2 (Bcl-2) and B-cell lymphoma-extra large (Bcl-xl) decreased, and the Bcl-xl ratio increased. These findings confirm the induction of apoptosis via the mitochondrial-dependent pathway [40]. In studies of human prostate cancer cells, treatment with BA similarly induced mitochondrial apoptosis. This induction manifests as alterations in the Bcl-2 ratio, increased cytochrome C release, caspase activation, and PARP cleavage, ultimately leading to cell death [41]. Furthermore, BA can synergistically induce apoptosis when combined with other therapeutic agents. In human hepatocellular carcinoma cells, the combination of BA and tumour necrosis factor-related apoptosis-inducing ligand (TRAIL) significantly enhances apoptosis induction. The mechanism underlying this synergistic effect involves upregulating p53 expression, thereby increasing caspase-3 cleavage and modulating the balance of Bcl-2 family proteins, manifested as downregulation of the antiapoptotic proteins Bcl-2 and Mcl-1 and upregulation of the proapoptotic proteins Bad and Bak [42]. This synergistic approach provides a promising strategy for enhancing the pro-apoptotic effects of BA in clinical settings.

Moreover, in addition to its direct action on mitochondria, BA can indirectly induce apoptosis by regulating other signalling pathways. For instance, in studies of human cervical cancer cells (HeLa), BA promotes ROS generation by inhibiting the expression of molecules in the PI3K signalling pathway, thereby triggering the mitochondrial apoptosis pathway [35]. Similarly, in studies of human hepatocellular carcinoma cells, the combination of BA and TRAIL synergistically induced apoptosis by upregulating p53 expression, increasing caspase-3 activation, and modulating the balanced expression of Bcl-2 family proteins [42]. These studies indicate that BA can both directly activate the mitochondrial apoptosis pathway and indirectly promote apoptosis by modulating other signalling pathways. This pro-apoptotic action of multiple pathways provides a crucial mechanistic basis for its antitumour effects [43].

### Mechanism through which BA inhibits epithelial–mesenchymal transition

EMT is a pivotal process that drives tumour invasion and metastasis, and BA effectively inhibits this pathway. In studies using SNU-16 gastric cancer cells, BA suppressed cellular migration and invasion. Western blot analysis indicated that this effect correlated with EMT inhibition, which was specifically manifested by the downregulation of the expression of EMT-associated marker proteins [44]. In SKOV3 ovarian cancer cells, BA similarly reduces cellular migration and invasive capacity by inhibiting the EMT process.

**Fig. 1:**
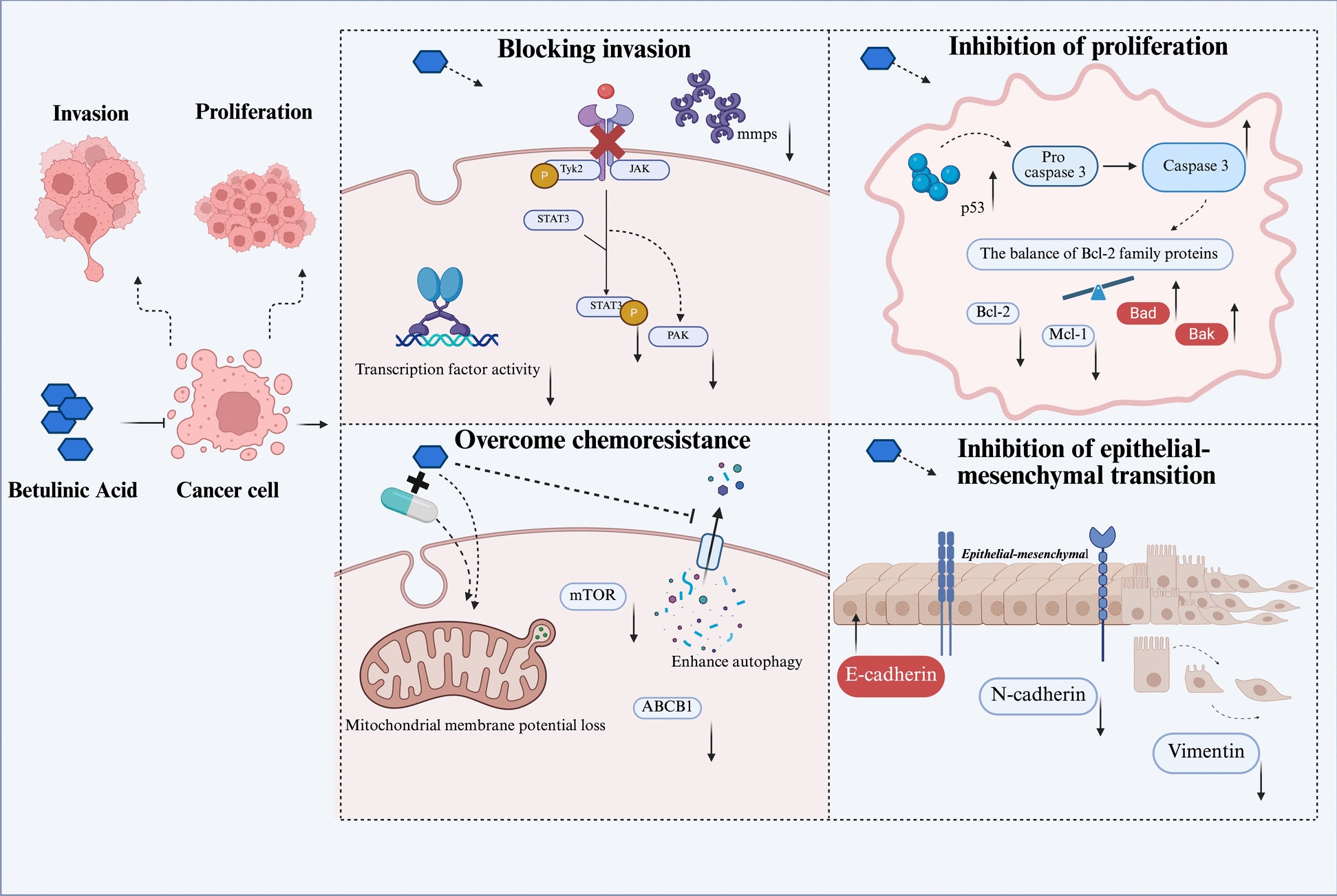
Mechanisms underlying the anti-tumour effects of BA

At the molecular level, BA inhibits EMT by modulating specific signalling pathways. For instance, in renal carcinoma cell studies, BA treatment downregulates the expression levels of Snail family transcriptional repressor 1 and Syndecan-2 in EMT-induced renal carcinoma cells while simultaneously suppressing cellular migration and invasive capabilities [45]. Moreover, in studies of non-small cell lung cancer, BA not only reduced the proportion of M2 tumour-associated macrophages by inhibiting the mTOR signalling pathway but also concurrently suppressed the EMT process, thereby diminishing the migratory and invasive capabilities of tumour cells [46]. These findings confirm that BA effectively inhibits the epithelial–mesenchymal transition process in tumour cells by regulating EMT-associated signalling pathways and the expression of effector proteins, thereby suppressing their invasive and metastatic capabilities [47]. The relevant mechanisms by which the above BA may exert anti-tumour efficacy are shown in Fig. 1.

## Mechanisms underlying the anti-inflammatory effects of BA

### BA inhibits classical inflammatory signalling pathways

**Fig. 2:**
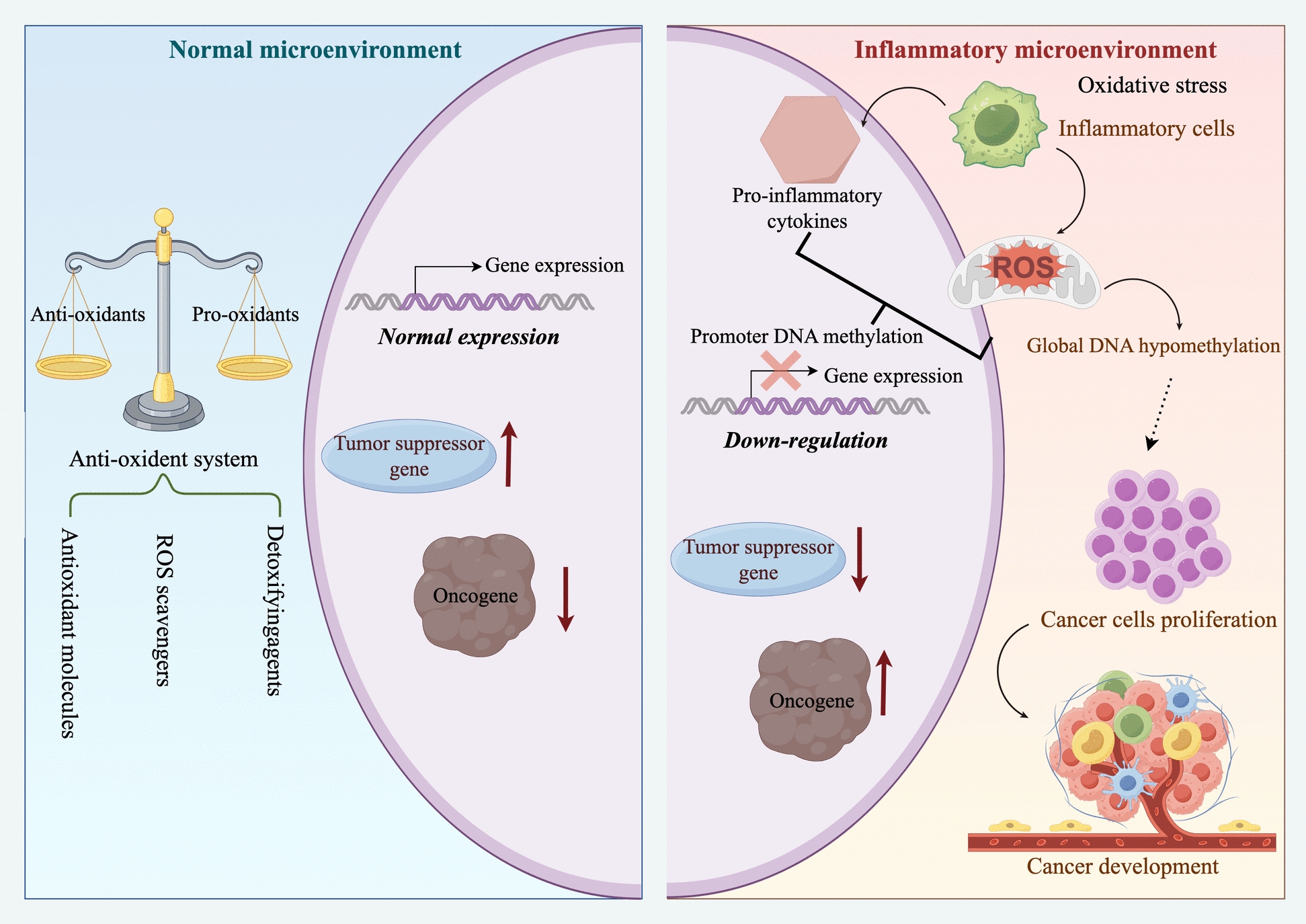
In the inflammatory microenvironment, inflammatory factors can promote the occurrence and development of tumours

The role of inflammation in tumour initiation, progression and metastasis has been extensively studied and widely recognised. Inflammation not only constitutes a fundamental characteristic of the tumour microenvironment but also directly promotes tumour progression through multiple mechanisms [48, 49]. Under physiological conditions, the anti-oxidant defence system maintains a dynamic balance between pro-oxidants and anti-oxidants, so that transiently generated ROS are efficiently removed and tumour suppressor genes and tumour-suppressive microRNAs remain normally expressed. By contrast, in a chronic inflammatory microenvironment, infiltrating inflammatory cells continuously produce ROS and pro-inflammatory cytokines such as IL-6. This oxidative and inflammatory stress transcriptionally up-regulates DNA methyltransferase 1, leading to promoter CpG hypermethylation and silencing of tumour suppressor genes and microRNAs, while excessive ROS simultaneously induce global DNA hypomethylation and genomic instability [48]. These inflammation-related genetic and epigenetic alterations favour oncogene activation and clonal expansion of transformed cells, thereby providing a mechanistic link between chronic inflammation and tumour initiation and progression, as schematically summarised in Fig. 2. For instance, chronic low-grade inflammation induced by obesity is a recognised carcinogenic risk factor. Dysfunctional adipose tissue secretes Tumour Necrosis Factor-alpha (TNF-α), Interleukin-6 (IL-6) and Interleukin-1 beta (IL-1β), and these proinflammatory cytokines form a microenvironment conducive to tumour growth through the induction of insulin resistance and oxidative stress [50]. Moreover, tumour cells themselves can actively sustain and amplify inflammatory states while also communicating with their surroundings through the secretion of extracellular vesicles (EVs) to promote inflammation and tumour metastasis. For instance, colorectal cancer cells secrete extracellular vesicles that encapsulate miR-99a-5p, activating the Nuclear Factor Kappa-light-chain-enhancer of activated B (NF-κB) cells signalling pathway in stromal fibroblasts and transforming them into cancer-associated fibroblasts (CAFs). This enhances the migratory capacity of colorectal cancer cells, subsequently inducing EMT via the C–C chemokine receptor type 5-mechanistic target of rapamycin–p70 ribosomal protein S6 kinase pathway, thereby accelerating tumour metastasis [51]. The inflammatory state within the tumour microenvironment may even directly influence treatment efficacy. For instance, tumour-associated neurotoxicity has been demonstrated to correlate with tumour burden in certain lymphoma patients [52].

BA markedly inhibits classical inflammatory signalling pathways such as the NF-κB pathway [53]. At the molecular level, the precise mechanism by which BA modulates these inflammatory pathways involves multi-target interactions. Molecular docking studies have revealed significant binding affinity and multiple interactions between BA and the ligand-binding domain of peroxisome proliferator-activated receptor gamma (PPAR-γ) [77]. Upon binding, BA activates the PPAR-γ signalling axis, which in turn suppresses NF-κB transcriptional activity through a reciprocal inhibitory mechanism. Specifically, PPAR-γ activation promotes the expression of the inhibitory protein IκBα, thereby preventing p65 nuclear translocation and reducing the transcription of downstream pro-inflammatory genes including TNF-α, IL-6, and COX-2. This PPAR-γ-mediated suppression of NF-κB represents a key molecular mechanism through which BA achieves its broad-spectrum anti-inflammatory effects. Additionally, BA’s activation of AMP-activated protein kinase (AMPK) connects its anti-inflammatory activity to metabolic reprogramming. Through AMPK activation, BA upregulates lipid oxidation-related genes (e.g., CPT1, ACOX1) while concurrently downregulating lipogenesis-associated genes (e.g., FAS, ACC), thus shifting the cellular metabolic profile away from a pro-inflammatory state. This metabolic reprogramming not only alleviates inflammation-associated metabolic disorders such as NAFLD but may also contribute to the anti-tumour microenvironment by reducing inflammation-driven tumour promotion [82]. In studies of RAW 264.7 macrophages stimulated with lipopolysaccharide (LPS), a methanol extract containing BA inhibited nitric oxide (NO) production and the expression of inducible nitric oxide synthase (iNOS) while simultaneously reducing the expression of proinflammatory cytokines such as interleukin IL-6, IL-1β, and tumour necrosis factor TNF-α at both the mRNA and protein levels [56]. Furthermore, in studies involving rheumatoid arthritis fibroblast-like synovial cells, BA inhibited TNF-α-induced cell proliferation, migration, and invasion. Concurrently, it suppressed the activation of the NF-κB signalling pathway, thereby reducing the production of inflammatory cytokines [57]. These findings clearly demonstrate that BA exerts anti-inflammatory effects across multiple pathological conditions through the inhibition of several core inflammatory signalling pathways. Given the foundational and driving role of inflammation in tumorigenesis and progression, we hypothesise that the potent anti-inflammatory properties of BA may represent a key pathway through which it achieves its anti-tumour efficacy. Elucidating the mechanism underlying its anti-inflammatory and anti-tumour effects represents a highly valuable avenue for future research.

### BA modulates immune cell function and polarisation

BA influences inflammatory and immune responses by regulating the function and polarisation state of immune cells. Within macrophages, it inhibits the production of nitric oxide and prostaglandin E_2_ induced by LPS while downregulating the expression levels of key proinflammatory factors such as TNF-α, IL-6, and IL-1β [58]. Furthermore, BA can guide macrophages to polarise from the proinflammatory M1 phenotype towards the anti-inflammatory M2 phenotype, thereby helping to alleviate excessive inflammatory responses and promoting tissue repair [59]. In terms of adaptive immunity, the BA derivative SH479 has a regulatory effect on T-cell subsets. It suppresses the differentiation of T helper 17 cell (Th17) cells while promoting the generation of regulatory T cells, thereby effectively correcting the imbalance in the Th17 ratio. In an experimental autoimmune encephalomyelitis mouse model, SH479 significantly ameliorated both clinical manifestations and histopathological damage. Mechanistically, SH479 impedes Th17 differentiation by inhibiting Signal Transducer and Activator of Transcription 3 (STAT3) phosphorylation and binding to the IL-17a promoter while simultaneously activating Signal Transducer and Activator of Transcription 5 (STAT5) and suppressing the NF-κB signalling pathway, thereby jointly regulating immune homeostasis [60]. Additionally, in studies of human peripheral blood lymphocytes, self-assembled BA enhances immunomodulatory activity. It not only promotes the proliferation of Cluster of Differentiation 4 positive T cells (CD4 + T) cells but also enhances the cytotoxic capacity of macrophages against tumour cells [61]. These studies indicate that BA interferes with inflammation by directly regulating the function, polarisation state, and associated signalling pathways of immune and adaptive immune cells, suggesting that it may exert an indirect anti-tumour effect by reversing immunosuppressive states.

### BA counteracts oxidative stress and inhibits inflammatory mediators

The anti-inflammatory effects of BA are also closely linked to its potent ability to suppress antioxidant stress and inflammatory mediators. In studies of experimental membranous nephropathy, BA significantly ameliorated renal oxidative stress states, manifested by reducing malondialdehyde levels, restoring antioxidant enzyme activity, and modulating gene and protein expression within the NF-κB and Nuclear factor erythroid 2-related factor 2 (Nrf2) signalling pathways. This response includes the downregulation of NF-κB, iNOS, and TNF-α expression and the upregulation of Nrf2, HO-1 (Heme Oxygenase-1), and NAD(P)H Quinone Dehydrogenase 1 expression, thereby mitigating renal inflammation and pathological damage [62].

**Fig. 3:**
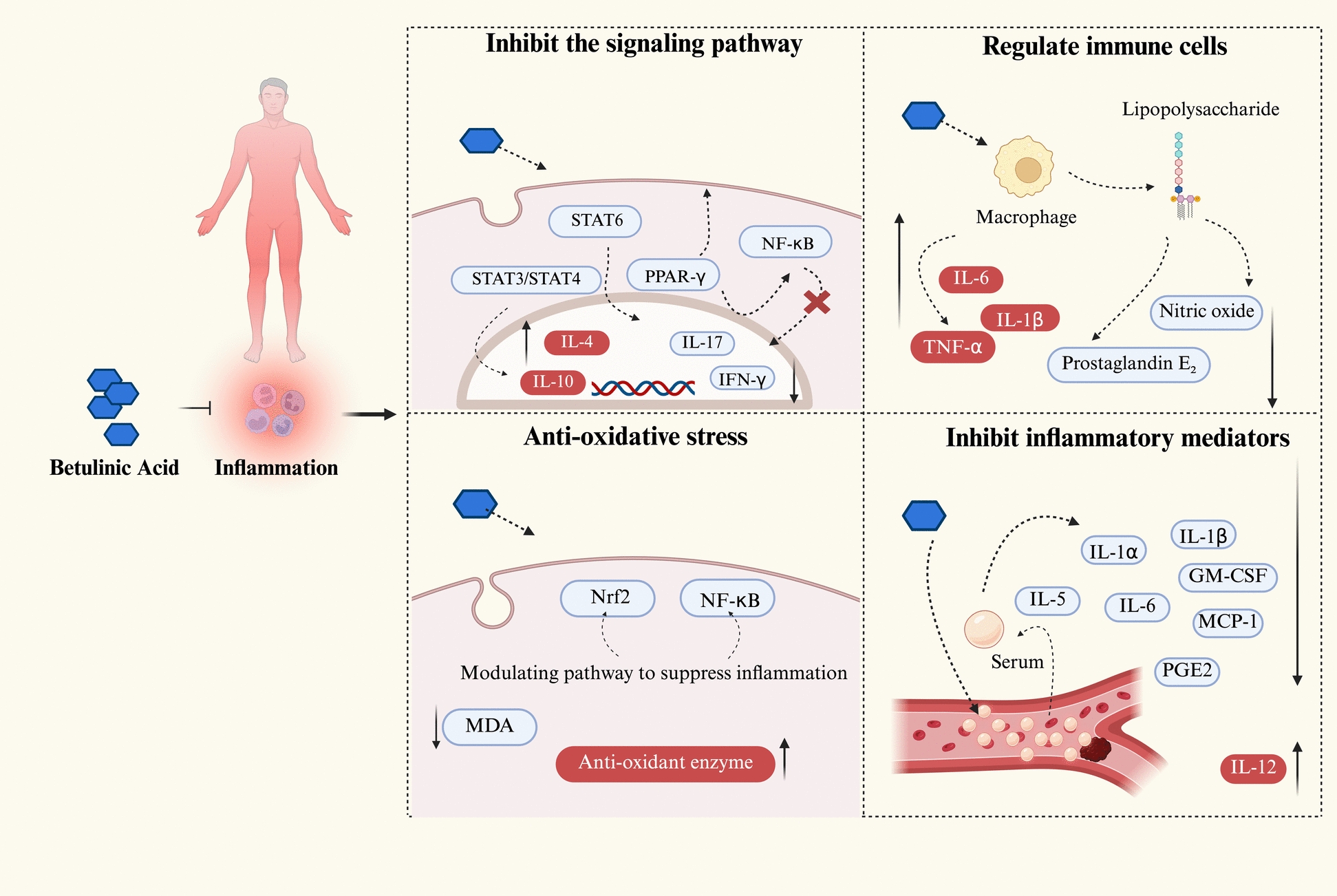
Mechanisms underlying the anti-inflammatory effects of BA

In studies across multiple inflammatory models, BA has been shown to have broad-spectrum inhibitory effects on inflammatory mediators. For instance, in a carrageenan-induced mouse paw oedema model, pretreatment with BA significantly reduced serum levels of Interleukin-1 alpha (IL-1α), IL-1β, Interleukin-5 (IL-5), IL-6, Granulocyte–macrophage colony-stimulating factor (GM-CSF), Monocyte chemoattractant protein-1 (MCP-1) and Prostaglandin E_2_ (PGE_2_) while simultaneously increasing the concentration of the anti-inflammatory cytokine Interleukin-12 [63]. Furthermore, BA inhibits neutrophil infiltration, reduces COX-2 protein expression, and decreases the phosphorylation of JNK, p38, and ERK1/2, indicating that its anti-inflammatory effects are mediated through suppression of the MAPK–COX-2–PGE2 signalling pathway. In studies of cerebral ischaemia–reperfusion injury, BA reduces ROS generation by downregulating NADPH oxidase 4 (NOX4) expression, thereby inhibiting neuronal apoptosis and mitigating brain damage [64]. These studies indicate that BA exerts crucial protective effects on various inflammation-related diseases by counteracting oxidative stress, activating endogenous antioxidant pathways, and suppressing the production of inflammatory mediators. The mechanisms involved in the possible anti-inflammatory effects of BA mentioned above are shown in Fig. 3.

## Therapeutic advances of BA in different tumours

The sustained interest in the antitumour properties of BA is intrinsically linked to its demonstrated potential therapeutic value across multiple tumour types. The antitumour activity of BA exhibits broad-spectrum efficacy, revealing promising therapeutic potential in a diverse range of neoplastic conditions.

### Leukaemia

In leukaemia research, BA has demonstrated a unique mechanism of action and therapeutic potential across multiple cancer treatments. In leukaemia treatment research, self-assembled nanoscale BA exhibits selective antileukaemic efficacy against the human leukaemia cell lines KG-1A and K562. It promotes cell death via ROS mediated pathways and increases proinflammatory cytokines—particularly by enhancing TNF-α-mediated cell death—while it is not significantly toxic to normal blood cells [65]. Moreover, BA demonstrates remarkable efficacy in reversing drug resistance. By promoting the ubiquitin-mediated degradation of Histone Deacetylase 3, it restores the sensitivity of drug-resistant chronic myeloid leukaemia cells to imatinib, offering a novel approach for addressing clinical drug resistance challenges [66].

### Ovarian cancer

When combined with existing anti-tumour drugs, BA also has remarkable synergistic effects. With respect to ovarian cancer treatment, the sequential administration of BA followed by 5-fluorouracil significantly induced apoptosis in OVCAR 432 cells, resulting in a cell growth inhibition rate exceeding 72%. These findings indicate that this combination therapy has significant application value in ovarian cancer treatment [67].

### Pancreatic cancer

In pancreatic cancer treatment, BA has also demonstrated multifaceted anti-tumour activity. When combined with gemcitabine, BA has synergistic pharmacological effects on the BxPC-3 pancreatic cancer cell line and the H1299 non-small cell lung cancer cell line, enhancing cell viability inhibition, apoptosis induction, and DNA double-strand breaks. BA enhances the efficacy of gemcitabine by promoting Chk1 degradation [68]. In the pancreatic cancer cell lines MIA PaCa-2 and PANC-1, the combined application of BA with either miR-101 or miR-24–2 reduced cell viability by 37–50%, indicating a synergistic effect. This concurrently influenced pro-caspase3, PARP cleavage, and Pyruvate Kinase M2 expression, thereby inducing apoptosis [69]. Furthermore, the combination of BA with sorafenib in non-small cell lung cancer and pancreatic ductal adenocarcinoma enhances the induction of apoptosis in cancer cells, promotes cell cycle arrest, and inhibits colony formation [70, 71].

### Colorectal cancer

In colorectal cancer, BA not only promotes cancer apoptosis but also inhibits cancer cell metastasis. It significantly suppressed tumour growth in a xenograft mouse model and is promising as a potential natural therapeutic agent for treating colorectal cancer [72]. Furthermore, in a study on the human colon cancer cell line HT-29, a novel BA analogue, 2c, was shown to induce apoptosis via a mitochondrial-dependent pathway. This manifested as increased ROS production, mitochondrial membrane potential depolarisation, activation of caspase-3 and caspase-9, PARP cleavage, nuclear degradation, and alterations in proapoptotic and antiapoptotic protein expression, with an IC_50_ value of 14.9 μM and negligible cytotoxicity towards normal peripheral blood mononuclear cells [40].

### Breast cancer

In breast cancer cells, BA and its derivatives can induce apoptosis and inhibit cell migration by activating apoptosis-related genes. For instance, BA and betulin derivatives extracted from Hibiscus syriacus activate p21 expression, induce cell cycle arrest, and simultaneously activate apoptosis-related genes. This inhibits the viability and migration of human triple-negative breast cancer cells while exhibiting minimal cytotoxicity towards normal mammalian epithelial cells [73].

### Gastric cancer

**Fig. 4:**
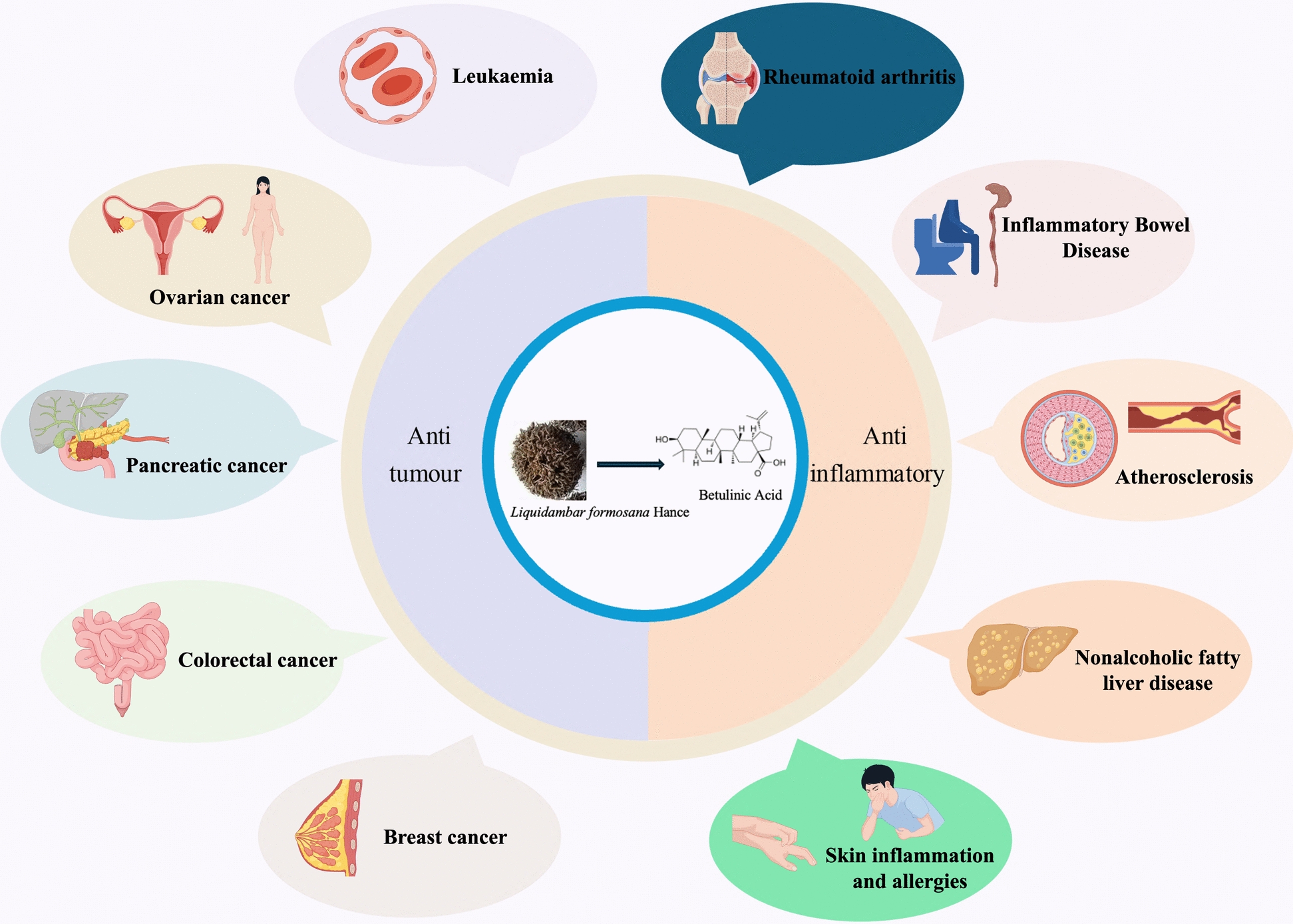
The disease types for which BA mainly exerts anti-tumour and anti-inflammatory effects

The mechanism by which betulonic acid exerts anti-gastric cancer effects may involve regulating the remodeling of the actin cytoskeleton in gastric cancer cells, influencing the expression levels of proteins related to the epithelial-mesenchymal transition signaling pathway, thereby inhibiting the invasion and metastasis of gastric cancer cells. At the same time, betulonic acid can also affect the expression levels of proteins related to matrix metalloproteinases in the lysates of gastric cancer cells [33]. The tumour types and mechanisms by which the above BA exerts anti-tumour effects are shown in Table 1 and Fig. 4.

**Table 1:** Therapeutic mechanisms of BA combined with chemotherapy agents in different tumours

Tumour type	The therapeutic mechanism of betulinic acid	References	Dosage and safety
Leukaemia	Self-assembled nanoscale BA induces ROS/TNF-α-mediated cell death without toxicity to normal blood cells	[65]	IC_50_ (cancer cells): PBL: 24 h: 159.62 µg/ml; KG-1A: 24 h: 14.87 µg/ml; K562: 24 h: 17.59 µg/ml Effect on normal cells: SA-BA showed no relevant toxicity to normal cells In vivo safety evaluation: Not reported
Ovarian cancer	Sequential administration of BA + 5-FU achieved an inhibition rate exceeding 72% Significantly induced apoptosis in OVCAR 432 cells	[67]	IC_50_ (cancer cells): Not reported Effect on normal cells: No relevant toxicity to normal cells reported In vivo safety evaluation: Not reported
Pancreatic cancer	Synergises with gemcitabine to promote Chk1 degradation Significantly inhibits tumour growth	[68]	IC_50_ (cancer cells): Not reported Effect on normal cells: No relevant toxicity to normal cells reported In vivo safety evaluation: Not reported
Colorectal cancer	Analogue 2c: IC_50_ = 14.9 µM, induces apoptosis and significantly inhibits tumour growth in mouse models	[40]	IC_50_ (cancer cells): HT-29:14.9 µM Effect on normal cells: low toxicity and high safety profile in normal cell lines In vivo safety evaluation: Not reported
Breast cancer	Induction of G0/G1 phase arrest, upregulation of p53/p21	[73]	IC_50_ (cancer cells): Not reported Effect on normal cells: No relevant toxicity to normal cells reported In vivo safety evaluation: Not reported
Gastric cancer	Inhibition of EMT and metastasis via regulation of actin cytoskeleton remodeling	[33]	IC_50_ (cancer cells): AGS: 24 h: 89.82 µM, 48 h: 38.33 µM, 72 h: 27.43 µM; HGC-27: 24 h: 75.06 µM, 48 h: 39.4 µM, 72 h: 33.47 µM Effect on normal cells: GES-1: 24 h: 120.84 µM, 48 h: 83.93 µM, 72 h: 73.56 µM. Smaller cytotoxic effects In vivo safety evaluation: Not reported

## The application of BA in inflammation-related diseases

### Rheumatoid arthritis

Rheumatoid arthritis (RA) is a chronic autoimmune inflammatory disease, and BA and its derivatives have demonstrated promising potential in its treatment.

At the cellular level, BA inhibits rheumatoid arthritis fibroblast-like synovial (RA-FLS) cells. Research has indicated that BA effectively suppresses the proliferation, migration, and invasive capacity of RA-FLS cells while concurrently reducing the mRNA expression levels of the TNF-α-induced inflammatory cytokines IL-1β, IL-6, IL-8, and IL-17A. Its mechanism of action is associated with inhibiting the activation of the NF-κB signalling pathway and diminishing the nuclear accumulation of NF-κB, thereby alleviating synovial inflammation [74].

In animal models, BA and its derivatives have also demonstrated favourable therapeutic effects. The BA derivative SH479 significantly suppressed T helper 1 cell (Th1) and Th17 cell polarisation, antigen-specific T-cell proliferation, and spleen lymphocyte-induced osteoclastogenesis in a collagen-induced arthritis (CIA) mouse model. This effectively reduced arthritis scores while mitigating bone destruction and cartilage depletion. Concurrently, this derivative modulates cytokine balance by reducing the levels of the proinflammatory cytokines IL-17 and Interferon-gamma (IFN-γ) while upregulating the anti-inflammatory cytokines IL-10 and Interleukin-4 IL-4. It ameliorates CIA symptoms through regulation of the Janus kinase-Signal Transducer and Activator of Transcription (JAK-STAT) signalling pathway [75]. Furthermore, when it is combined with fluvastatin, BA significantly reduces the arthritis index and the levels of rheumatoid factor, C-reactive protein, total lipid, and anti-cyclic citrullinated peptide antibodies in CIA mice. It reduced proinflammatory enzyme activity and oxidative stress in peripheral blood mononuclear cells, increased the expression of the anti-inflammatory cytokine IL-10, and effectively decreased the risk of RA and cardiovascular disease by modulating the activity of the Toll-like receptor-4–NF-κB downstream signalling pathway [76].

### Inflammatory bowel disease

Inflammatory bowel disease (IBD), including ulcerative colitis (UC) and Crohn’s disease (CD), has demonstrated favourable therapeutic outcomes with mesalamine.

In UC animal models, BA effectively alleviates disease symptoms. Taking a dextran sulfate sodium (DSS)-induced mouse UC model as an example, oral administration of BA significantly prevents diarrhoea, haemorrhage, and pathological changes in the colon. It reduces the levels of nitrite, malondialdehyde, myeloperoxidase, and lipid hydroperoxide in colonic tissue. It restored superoxide dismutase and catalase activity and reduced glutathione levels to normal levels, effectively regulating redox balance. Concurrently, BA reduces the levels of inflammatory mediators such as matrix metalloproteinase-9 and prostaglandin E2, thereby mitigating intestinal inflammatory responses [54].

BA also exerts therapeutic effects by modulating relevant signalling pathways. In DSS-induced IBD mice and in vitro cell and organoid models, BA ameliorated weight loss and colon shortening, reduced disease activity index scores, and diminished histopathological damage to colonic tissue. In vitro experiments demonstrated that BA reduces FITC-dextran flux, increases transepithelial electrical resistance, and decreases the production of the proinflammatory cytokines IL-6, IL-1β and TNF-α while simultaneously increasing the mRNA expression levels of the anti-inflammatory cytokine IL-10. Furthermore, BA enhances intestinal epithelial barrier formation by upregulating the expression of the tight junction proteins zonula occludens-1 (ZO-1), occludin, and claudin-1. Molecular docking studies revealed significant docking scores and interactions between BA and peroxisome proliferator-activated receptor gamma (PPAR-γ). By modulating the PPAR-γ/NF-κB signalling pathway, it inhibits inflammatory responses and enhances tight junction protein expression, thereby alleviating IBD symptoms [77].

Moreover, novel formulations of BA have demonstrated therapeutic potential. A multifunctional diagnostic and therapeutic platform based on polymeric nanoparticles loaded with BA and resveratrol enables preferential targeting to inflamed colonic regions. Utilising bioluminescence and fluorescence resonance energy transfer effects to facilitate visual monitoring of tissue inflammation, BA significantly alleviated UC symptoms and did not disrupt gut microbiota homeostasis or cause organ damage or other side effects [78].

### Skin inflammation and allergic conditions

In the treatment of skin inflammation and allergic conditions, BA has demonstrated favourable therapeutic effects.

With respect to ultraviolet radiation-induced psoriasis-like wounds, topical treatment with standardised BA extracted from Dillenia indica fruit effectively accelerated the wound healing process. Research has indicated that the extract neutralises lipid peroxidation at an in vitro concentration of 0.02 μg/mL and significantly accelerates wound healing at 50 mg/mL. Notably, the ethyl acetate extract demonstrated efficacy comparable to that of the positive control clobetasol, reducing complete wound healing time while decreasing hyperkeratinisation and providing superior protection against skin biomolecular oxidation [79].

In a psoriasis mouse model, BA effectively alleviated disease symptoms. It reduces the Psoriasis Area and Severity Index (PASI) score; decreases epidermal thickness and T-cell infiltration; decreases the frequency of IL-17A-expressing CD4⁺ and γδ T cells; increases the levels of the anti-inflammatory cytokine IL-10; and inhibits the proinflammatory mediators Retinoic acid receptor-related orphan receptor gamma (RORγt), IL-17A, IL-6, and TNFα in skin lesions while inhibiting NF-κB signalling pathway activation. Furthermore, BA suppresses T-cell proliferation and IL-17A production in CD4⁺ T cells, thereby mitigating the inflammatory response in psoriasis [80].

Nanoparticle formulations of BA also demonstrate advantages in treating skin conditions. When prepared as nanogels or loaded onto solid lipid nanoparticles before incorporation into hydrogel matrices, BA can be employed to treat psoriasis-like inflammatory skin disorders. Compared with conventional gels and free BA, the nanogel formulation demonstrated potent anti-inflammatory activity in a mouse ear oedema model, achieving a 52% reduction in oedema [81]. The BA–solid lipid nanoparticle–hydrogel formulation exhibited favourable drug release characteristics and skin permeation properties in vitro. In a mouse model of psoriasis-like skin inflammation induced by imiquimod, it significantly alleviated inflammatory severity and reduced the levels of associated cytokines, demonstrating promising therapeutic efficacy [55].

### Nonalcoholic fatty liver disease

Nonalcoholic fatty liver disease (NAFLD) is a prevalent chronic liver disorder classified as an inflammation-associated metabolic condition, with BA demonstrating significant therapeutic potential in its management.

By activating relevant signalling pathways, BA may improve the pathological progression of NAFLD. Research has indicated that BA activates AMP-activated protein kinase (AMPK), effectively reducing weight gain and tissue fat deposition in a high-fat diet-induced obese mouse model without altering total caloric intake. Gene expression profiling revealed that BA treatment upregulated the expression of genes related to lipid oxidation but downregulated the expression of genes associated with lipogenesis. By promoting energy expenditure, enhancing lipid oxidation, and increasing thermogenic capacity, it exerts protective effects against obesity and NAFLD [82].

Furthermore, BA can improve NAFLD through the modulation of other signalling pathways. In NAFLD mouse models, BA inhibits the expression and activity of fatty acid synthase (FAS). Transcriptional suppression of Yin Yang 1 (YY1) reduces abnormal hepatic triglyceride accumulation and mitigates fatty acid synthesis, hepatic fibrosis and inflammatory responses while simultaneously promoting fatty acid oxidation. This protects hepatocytes from damage caused by abnormal lipid deposition [83]. Notably, BA also functions as an agonist of the farnesoid X receptor (FXR), activating the FXR signalling pathway. This inhibits the activity of the intracellular PERK/EIF2α/ATF4 and CHOP signalling pathways, alleviating endoplasmic reticulum stress in the liver. It effectively mitigates the progression of NAFLD and metabolic disorders while restoring endoplasmic reticulum homeostasis in hepatocytes. In FXR gene knockout mice, the protective effects of BA are markedly diminished, further confirming the critical role of the FXR pathway in its mechanism of action [84].

### Atherosclerosis

Atherosclerosis is a chronic inflammatory disease in which BA plays a significant role in its prevention and treatment [85].

In animal models, BA clearly protects against atherosclerosis. In diabetic apolipoprotein E knockout mice, 12 weeks of BA treatment significantly reduced systolic blood pressure; improved metabolic parameters; lowered blood urea nitrogen, triglyceride and total cholesterol levels; and improved glucose, insulin, and glucose tolerance outcomes and the insulin resistance index. Concurrently, BA treatment reduced atherosclerotic lesion formation, increased endothelial nitric oxide synthase (eNOS) expression, and suppressed intercellular adhesion molecule-1 (ICAM-1) and endothelin-1 (ET-1) expression. These findings suggest that BA may play a significant role in the treatment and prevention of early atherosclerosis by alleviating endothelial dysfunction.

Moreover, BA exerts antiatherosclerotic effects through additional mechanisms. It performs vasoprotective functions by activating calcium signalling pathways, thereby enhancing eNOS phosphorylation and nitric oxide synthesis. In certain combination therapy studies, BA administered alongside fluvastatin demonstrated efficacy against rheumatoid arthritis-associated atherosclerosis. This was achieved by modulating the expression of the Toll-like receptor-4–NF-κB downstream signalling pathway, thereby reducing inflammatory mediator expression, lowering atherosclerosis indices, and mitigating oxidative stress levels [76]. The types of inflammation and related mechanisms by which the above BA exerts anti-inflammatory effects are shown in Table 2 and Fig. 4.

**Table 2:** BA exerts anti-inflammatory effects

Disease type	Research model	Primary pharmacological effects	Mechanism of action	References	Dosage and safety
Rheumatoid arthritis (RA)	In vitro RA-FLS cells	Inhibits cell proliferation, migration, and invasion; reduces expression of IL-1β, IL-6, IL-8, and IL-17A	Inhibits the NF-κB signalling pathway, reducing NF-κB nuclear translocation	[74]	IC_50_ (target cells): Not reported Effect on normal cells: Not reported In vivo safety evaluation: Not explicitly reported
Inflammatory Bowel Disease (IBD)	DSS-induced mouse ulcerative colitis model	Alleviates diarrhoea, haemorrhage, and pathological changes in the colon; reduces oxidative stress and levels of inflammatory mediators	Regulates redox balance, reduces MMP-9 and PGE2; enhances expression of tight junction proteins (ZO-1, occludin, etc.)	[54, 77]	IC_50_ (target cells): Not reported Effect on normal cells: Not reported In vivo safety evaluation: Not explicitly reported
	Cell and organoid models	Enhances intestinal barrier function, reduces proinflammatory factors (IL-6, IL-1β, TNF-α), and elevates IL-10	Activates the PPAR-γ/NF-κB pathway to suppress inflammation and enhances barrier integrity		
Skin inflammation and allergies	Psoriasis mouse model	Reduces PASI scores, decreases epidermal thickness and T-cell infiltration, and inhibits IL-17A expression	Inhibits the NF-κB and RORγt pathways to reduce the expression of proinflammatory factors	[80]	IC_50_ (target cells): T cell: 48 h: 18.17 µM, 96 h: 9.24 µM Effect on normal cells: Not reported In vivo safety evaluation: Not explicitly reported
Nonalcoholic fatty liver disease	High-fat diet-fed obese mice	Reduces body weight and fat deposits, improves hepatic lipid metabolism	Activates AMPK to promote lipid oxidation and inhibits fat formation	[82–84]	IC_50_ (target cells): Not reported Effect on normal cells: Not reported In vivo safety evaluation: Not explicitly reported
	NAFLD mouse model	Reduces hepatic triglyceride accumulation and attenuates inflammation and fibrosis	Inhibits the YY1/FAS pathway and promotes fatty acid oxidation		
	FXR⁻/⁻ mouse model	Alleviates endoplasmic reticulum stress and hepatic injury	Activates the FXR signalling pathway, inhibits the PERK/EIF2α/ATF4/CHOP pathway		
Atherosclerosis	ApoE knockout diabetic mice	Improves metabolic parameters, reduces atherosclerotic lesions, and enhances endothelial function	Upregulates eNOS, inhibits ICAM-1 and ET-1 expression	[85]	IC_50_ (target cells): Not reported Effect on normal cells: Not reported In vivo safety evaluation: Not explicitly reported
	Endothelial cells	Increases eNOS phosphorylation and NO synthesis	Activates the calcium signalling pathway		

## Synthesis and applications of BA derivatives

To improve the pharmacokinetic properties of BA and enhance its antitumour activity, researchers have optimised its synthesis and modification through multiple strategies [86]. To facilitate a comprehensive understanding of the structure–activity relationships discussed below, the chemical structures of BA and its key derivatives are presented in Table 3. The parent compound BA (3β-hydroxy-lup-20(29)-en-28-oic acid, C₃₀H₄₈O₃) features a lupane-type pentacyclic triterpenoid skeleton with three main modifiable sites: the C-3 hydroxyl group, the C-19 isopropyl group, and the C-28 carboxyl group. Structural modifications at these positions have yielded derivatives with substantially enhanced anti-tumour activity. For instance, heterocyclic modification (compound 17) at the C-28 position produced a derivative with approximately 20-fold higher activity than BA itself. The triazole-containing analogue (compound 2c) introduced a nitrogen-containing heterocycle that enhanced selectivity against colon cancer cells (HT-29, IC_50_ = 14.9 μM). The codrug Bet-CA, linking BA with dichloroacetic acid at C-28, achieved improved water solubility alongside synergistic cytotoxic effects via mitochondrial pathway activation. These structural modifications demonstrate that systematic chemical modification of the BA scaffold can effectively enhance its pharmacological profile while addressing its intrinsic limitations. With respect to biosynthetic mechanisms, functional studies of triterpenoid synthesis-related genes in birch revealed that inhibition of the Bpβ-AS gene positively regulates BA synthesis, whereas interference with the BpCAS gene promotes downstream BA production. These findings provide crucial theoretical foundations for enhancing BA yield and achieving targeted modifications through genetic engineering techniques [87]. These structure–activity relationships are comprehensively illustrated in Table 3, which presents the chemical structures, modification sites, and corresponding biological activities of BA and its key derivatives.

**Table 3:** Structure–activity relationships of BA and its key derivatives

Compound name	Chemical structure
Betulinic acid (BA) (Parent compound)	
Heterocyclically modified derivative (Compound 17)	
Triazole analogue (Compound 2c)	
C-28 ester derivatives (38-compound library)	
SYK023 (BA derivative)	
SH479 (BA derivative)	
Bet-CA (BA–dichloroacetic acid codrug)	
F-8arm-PEG-BA (Folate-PEG-BA nanoparticle)	

Within the field of chemical modification, researchers have successfully synthesised a series of BA derivatives. Among these derivatives, heterocyclically modified BA derivatives exhibit significant anti-tumour activity against multiple tumour cell lines. The most potent compound, 17, has an average IC_50_ of 1.19 μM—approximately 20-fold higher activity than BA itself. Its inhibitory effect on the multidrug-resistant tumour cell line MCF-7/ADR is notably 117 times greater than that of BA [88]. Another study synthesised triazole-containing BA analogues, among which Compound 2c exhibited outstanding inhibitory effects against the human colon cancer cell line HT-29 (IC_50_ value of 14.9 μM) and effectively induced apoptosis via a mitochondria-dependent pathway [40]. The synthesis and modification of these derivatives provide a robust chemical foundation for the development of more effective anti-tumour drugs.

In clinical research, BA derivatives have also demonstrated promising applications. A systematic study evaluated the anti-tumour activity of 38 BA ester derivatives modified at the C-28 position and revealed that multiple derivatives exhibited significant antiproliferative activity against human leukaemia MV4-11, A549 lung carcinoma, PC-3 prostate carcinoma, and MCF-7 breast carcinoma cell lines, with IC50 values ranging from 2 to 5 μM. Furthermore, some active compounds induce apoptosis by activating the caspase-3/7 pathway [89]. Another study reported that the novel BA derivative SYK023 effectively inhibited lung tumour proliferation in two distinct mouse models of lung cancer without observable significant side effects. This derivative induces apoptosis by triggering endoplasmic reticulum stress responses while substantially reducing the metastatic capacity of lung cancer cells [28]. These preclinical studies provide robust experimental evidence for the progression of BA derivatives into clinical trials.

Notably, substantial progress has been made in the development of BA derivatives as therapeutic agents. For instance, the codrug Bet-CA, which is composed of BA and dichloroacetic acid, not only has increased water solubility but also has significant synergistic cytotoxic effects on multiple cancer cell lines in vitro. This compound selectively eliminates cancer cells cocultured with human fibroblasts. Its mechanism of action involves inducing ROS production, altering the mitochondrial membrane potential, and releasing cytochrome c, ultimately triggering apoptosis via the mitochondrial pathway. In vivo studies have also demonstrated tumour-suppressive effects, with no significant toxic reactions observed within clinically achievable dosage ranges [90].

## Drug delivery systems, targeted therapy, and safety evaluation of BA and its derivatives

Targeted therapy represents a significant advancement in modern oncology, with BA and its derivatives demonstrating unique advantages in this field. Through rational structural design, certain BA derivatives can specifically target particular molecular markers on tumour cells, enabling precision treatment [91]. For instance, researchers have successfully engineered folate-conjugated octa-arm polyethylene glycol-betulinic acid (F-8arm-PEG-BA) nanoparticles for the codelivery of anti-tumour drugs. This approach significantly enhances drug accumulation at tumour sites, thereby increasing therapeutic efficacy [92]. To facilitate the clinical translation of BA, a comparative evaluation of different nano-delivery strategies is warranted. Currently, the main delivery systems for BA can be broadly categorised into natural carrier-based systems (e.g., chitosan nanoparticles, tumour cell membrane-coated carriers) and synthetic carrier-based systems (e.g., PLGA nanoparticles, PEGylated liposomes, solid lipid nanoparticles). Natural carriers generally offer superior biocompatibility and reduced immunogenicity, while synthetic carriers provide greater control over particle size, drug loading, and release kinetics. In terms of targeting, active-targeted systems such as folate-conjugated nanoparticles (F-8arm-PEG-BA) [91] and aptamer-modified systems demonstrate enhanced tumour accumulation and cellular uptake compared with passively targeted formulations that rely solely on the enhanced permeability and retention (EPR) effect. For example, a recent study demonstrated that a folate-coated micelles-in-liposomes system co-loaded with BA and celastrol achieved sequential drug release, targeting both cancer-associated fibroblasts and tumour cells to overcome drug resistance in triple-negative breast cancer models. However, each delivery system presents distinct trade-offs: liposomal formulations offer good biocompatibility but may suffer from drug leakage during circulation; polymeric nanoparticles achieve sustained release but face challenges in large-scale production consistency; nanogels provide localised delivery advantages for topical applications (e.g., psoriasis) but are less suitable for systemic administration; and carbon nanotube-based carriers demonstrate excellent drug loading capacity but raise long-term biosafety concerns due to their non-biodegradable nature. From a clinical feasibility perspective, liposomal and PLGA-based formulations are the most advanced, with established regulatory pathways for similar products, whereas emerging systems such as tumour cell membrane-coated nanoparticles, though promising, require further validation of manufacturing scalability and batch-to-batch reproducibility. Furthermore, certain BA derivatives exert therapeutic effects by targeting metabolic reprogramming processes in tumour cells. For instance, they inhibit aerobic glycolysis in tumour cells, thereby disrupting their energy metabolism pathways and suppressing tumour cell growth and proliferation [93]. This targeted strategy, which is based on the specific metabolic characteristics of tumour cells, offers novel approaches and methods for cancer treatment.

Presently, clinical research into BA for tumour treatment remains in its early stages, yet existing data indicate promising application potential. Although no large-scale, multicentre clinical trials have been reported, multiple preliminary studies have laid the groundwork for further clinical application. In several small-scale preclinical studies, BA and its derivatives have demonstrated favourable antitumour activity against multiple tumour cell lines and animal models but low toxicity towards normal cells. This provides a rationale for further translational studies and carefully designed early-phase clinical evaluation [94]. For instance, in melanoma research, the topical application of BA and its derivatives has demonstrated therapeutic efficacy against early-stage melanoma, with observable reductions in tumour volume and no significant severe adverse reactions observed [95]. Furthermore, in vitro studies on breast cancer cells demonstrated that a BA derivative significantly inhibited cell proliferation and induced apoptosis but exhibited minimal toxicity towards normal cells [73]. If applied clinically, this approach could theoretically offer a novel treatment option for patients with breast cancer, particularly those who are resistant to or intolerant of conventional therapies [96].

In animal studies, nanoliposomes loaded with BA demonstrated favourable therapeutic effects against colorectal cancer, effectively inhibiting tumour growth and metastasis [97]. These findings suggest that in clinical practice, such nanomedicines may emerge as novel therapeutic approaches for colorectal cancer. However, numerous challenges must be addressed before transitioning from laboratory research to clinical application, including optimising large-scale production processes, conducting long-term safety evaluations, and ensuring compatibility with existing clinical treatment regimens. Only by resolving these issues can BA derivatives be genuinely deployed in clinical settings, potentially expanding future therapeutic options for patients. BA therapy has certain advantages in terms of safety. Multiple studies have indicated that within a defined dosage range, BA has relatively low toxicity towards normal cells and tissues. In rat experiments, the administration of specific doses of BA resulted in no significant organ toxicity or mortality, with no marked effect on functional indicators of vital organs such as the liver and kidneys [98]. In studies of human peripheral blood lymphocytes, pretreatment with BA mitigated doxorubicin-induced cytotoxicity, protecting cells from oxidative stress and apoptosis. This finding suggests its potential as an adjuvant therapy to reduce the adverse effects of chemotherapy drugs [99]. However, some studies have indicated that at high doses or with prolonged use, BA may cause certain mild adverse reactions. For instance, in certain animal studies, high doses of BA may lead to minor gastrointestinal discomfort, such as diarrhoea, although these reactions typically resolve spontaneously after discontinuation of the medication [100]. Overall, available preclinical data suggest a relatively favourable safety profile for BA within tested dose ranges; however, systematic toxicology and human safety data are still required, and close monitoring would be essential in any clinical application [101].

## Discussion

This review provides a comprehensive overview of BA as a naturally occurring pentacyclic triterpenoid with dual anti-tumour and anti-inflammatory potential. Regarding its anti-tumour effects, BA targets multiple critical processes in tumour biology, including inhibition of tumour cell invasion (via STAT3/FAK axis and MMP/TIMP-2 regulation), suppression of tumour cell proliferation (through cell cycle arrest at the G0/G1 phase), reversal of chemotherapy resistance (independent of classical P-glycoprotein-mediated mechanisms), induction of mitochondrial apoptosis, and inhibition of the epithelial–mesenchymal transition. In the anti-inflammatory domain, BA exerts its effects through inhibition of the NF-κB signalling pathway, modulation of immune cell polarisation (M1-to-M2 macrophage shift and Th17/Treg balance), and counteraction of oxidative stress via the Nrf2/HO-1 pathway. These multi-target mechanisms underscore the therapeutic versatility of BA, particularly in the context of inflammation-associated cancers where anti-tumour and anti-inflammatory activities may synergistically contribute to improved outcomes.

The inherent pharmacokinetic limitations of BA, including poor aqueous solubility and low oral bioavailability, have driven the development of diverse nano-enabled drug delivery strategies. Among these, nanoliposomes, polymeric nanoparticles (PLGA-based), nanogels, solid lipid nanoparticles, and carbon nanotube-based systems have demonstrated varying degrees of improvement in BA’s therapeutic efficacy. Active targeting strategies, such as folate-conjugated and aptamer-modified nanoparticles, have shown enhanced tumour accumulation compared with passively targeted formulations. However, each delivery system presents distinct advantages and limitations in terms of biocompatibility, drug loading capacity, release kinetics, and clinical scalability, necessitating a careful selection based on the intended therapeutic application and route of administration.

The combination of BA with conventional chemotherapeutic agents (e.g., gemcitabine, 5-FU, sorafenib), immunotherapy agents, or targeted therapies has emerged as a promising strategy to achieve synergistic therapeutic effects and overcome tumour drug resistance. Moreover, given the heterogeneity of tumour cells across patients, BA’s ability to modulate multiple signalling pathways (e.g., PI3K/Akt, NF-κB, mTOR) positions it as a potential component of personalised treatment regimens, where the specific pathway profile of individual tumours could guide the selection of BA-based combination strategies. The development of BA derivatives through structural modification further expands the therapeutic options, with some derivatives demonstrating activity 20–117 times greater than that of the parent compound.

Despite the encouraging preclinical evidence, several critical challenges must be addressed before BA can be translated into clinical practice. First, the majority of studies are based on in vitro cell line models and rodent studies, with a notable absence of data from larger animal models or human clinical trials. The extrapolation of in vitro IC_50_ values to clinically achievable plasma concentrations remains uncertain. Second, while BA demonstrates immunomodulatory properties, the potential for immune-related adverse events, including autoimmune reactions or immunosuppression-related complications during long-term administration, has not been systematically evaluated. Third, the in vivo degradation kinetics and long-term fate of BA nanoformulations remain poorly understood; questions regarding nanocarrier biodistribution, organ accumulation, clearance pathways, and potential chronic toxicity of carrier materials (particularly non-biodegradable systems) require urgent investigation. Fourth, the transition from laboratory-scale preparation to large-scale manufacturing of BA nanoformulations poses significant challenges, including maintaining batch-to-batch consistency in particle size and drug loading, ensuring long-term storage stability, meeting sterilisation requirements, and navigating the regulatory frameworks for nanomedicine approval.

Looking ahead, several key research priorities merit attention. Further structural optimisation of BA derivatives through computer-aided drug design could yield compounds with improved potency, selectivity, and pharmacokinetic profiles. The identification of novel molecular targets, particularly those at the intersection of tumour metabolism and immune regulation, may reveal new therapeutic opportunities. The integration of artificial intelligence in nanomedicine design holds promise for accelerating the optimisation of drug delivery systems. Most critically, well-designed early-phase clinical trials are urgently needed to establish the safety, pharmacokinetics, and preliminary efficacy of BA and its most promising derivatives in human subjects. Such clinical data will be essential for determining the optimal dosing regimens, identifying potential biomarkers of response, and establishing the clinical positioning of BA within the existing armamentarium of anti-tumour and anti-inflammatory therapies.

## Data Availability

Data sharing is not applicable to this article as no datasets were generated or analysed during the current study.

## Abbreviations

BA: Betulinic acid
EMT: Epithelial mesenchymal transition
Sp1: Specific protein 1
TRAIL: Tumour necrosis apoptosis inducing ligand
MDA-MB-231: M.D. Anderson Cancer Center-Metastatic Breast Cancer 231
ROS: Reactive oxygen species
Bcl-2: B-cell lymphoma 2
IL-6: Interleukin-6
EVs: Extracellular vesicles
NF-κB: Nuclear factor Kappa-light-chain-enhancer of activated B cells
NO: Nitric oxide
Th17: T helper 17 cell
STAT5: Signal transducer and activator of transcription 5
Nrf2: Nuclear factor erythroid 2-related factor 2
IL-1α: Interleukin-1 alpha
GM-CSF: Granulocyte-macrophage colony-stimulating factor
PGE_2_: Prostaglandin E_2_
RA: Rheumatoid arthritis
Th1: T helper 1 cell
IL-17: Interleukin-17
IL-10: Interleukin-10
JAK-STAT: Janus kinase-signal transducer and activator of transcription
UC: Ulcerative colitis
DSS: Dextran sulfate sodium
PPAR-γ: Peroxisome proliferator-activated receptor gamma
RORγt: Retinoic acid receptor-related orphan receptor gamma
AMPK: AMP-activated protein kinase
YY1: Yin Yang 1
LPL: Lipoprotein lipase
ICAM-1: Intercellular adhesion molecule-1
F-8arm-PEG-BA: Folate-conjugated octa-arm polyethylene glycol-betulinic acid
TCM: Traditional Chinese medicine
DAPPI: Desorption atmospheric pressure photoionisation
MMP: Matrix metalloproteinase
PCNA: Proliferating cell nuclear antigen
PI3K: Phosphoinositide 3-kinase
MCF-7: Michigan Cancer Foundation-7
PARP: Poly Adenosine diphosphate-ribose polymerase
Bcl-xl: B-cell lymphoma-extra large
TNF-α: Tumour necrosis factor-alpha
IL-1β: Interleukin-1 beta
CAFs: Cancer-associated fibroblasts
LPS: Lipopolysaccharide
INOS: Inducible nitric oxide synthase
STAT3: Signal transducer and activator of transcription 3
CD4 + T: Cluster of differentiation 4 positive T cells
HO-1: Heme oxygenase-1
IL-5: Interleukin-5
MCP-1: Monocyte chemoattractant protein-1
NOX4: NADPH oxidase 4
RA-FLS: Rheumatoid arthritis fibroblast-like synovial
CIA: Collagen-induced arthritis
IFN-γ: Interferon-gamma
IL-4: Interleukin-4
IBD: Inflammatory bowel disease
CD: Crohn’s disease
ZO-1: Zonula occludens-1
PASI: Psoriasis area and severity index
NAFLD: Nonalcoholic fatty liver disease
FAS: Fatty acid synthase
FXR: Farnesoid X receptor
eNOS: Endothelial nitric oxide synthase
ET-1: Endothelin-1
